# IFNγ-stimulated dendritic cell extracellular vesicles can be nasally administered to the brain and enter oligodendrocytes

**DOI:** 10.1371/journal.pone.0255778

**Published:** 2021-08-13

**Authors:** Kae M. Pusic, Richard P. Kraig, Aya D. Pusic

**Affiliations:** Department of Neurology, The University of Chicago, Chicago, IL, United States of America; Instituto Cajal-CSIC, SPAIN

## Abstract

Extracellular vesicles secreted from IFNγ-stimulated rat dendritic cells (referred to here as IFNγ-DC-EVs) contain miRNAs which promote myelination (including but not limited to miR-219), and preferentially enter oligodendrocytes in brain slice cultures. IFNγ-DC-EVs also increase myelination when nasally administered to naïve rats. While we can infer that these extracellular vesicles enter the CNS from functional studies, here we demonstrate biodistribution throughout the brain after nasal delivery by way of imaging studies. After nasal administration, Xenolight DiR-labelled IFNγ-DC-EVs were detected 30 minutes later throughout the brain and the cervical spinal cord. We next examined cellular uptake of IFNγ-DC-EVs by transfecting IFNγ-DC-EVs with mCherry mRNA prior to nasal administration. mCherry-positive cells were found along the rostrocaudal axis of the brain to the brainstem. These cells morphologically resembled oligodendrocytes, and indeed cell-specific co-staining for neurons, astrocytes, microglia and oligodendrocytes showed that mcherry positive cells were predominantly oligodendrocytes. This is in keeping with our prior *in vitro* results showing that IFNγ-DC-EVs are preferentially taken up by oligodendrocytes, and to a lesser extent, microglia. To confirm that IFNγ-DC-EVs delivered cargo to oligodendrocytes, we quantified protein levels of miR-219 mRNA targets expressed in oligodendrocyte lineage cells, and found significantly reduced expression. Finally, we compared intranasal versus intravenous delivery of Xenolight DiR-labelled IFNγ-DC-EVs. Though labelled IFNγ-DC-EVs entered the CNS via both routes, we found that nasal delivery more specifically targeted the CNS with less accumulation in the liver. Taken together, these data show that intranasal administration is an effective route for delivery of IFNγ-DC-EVs to the CNS, and provides additional support for their development as an EV-based neurotherapeutic that, for the first time, targets oligodendrocytes.

## Introduction

Environmental enrichment consists of volitionally increased physical, social and intellectual activity. Environmental enrichment enhances memory, increases myelination at all ages, and has been shown to reduce the impact of neurodegenerative disorders including demyelinating diseases [1–4]. Work from our laboratory shows that exposure of rats to an environmental enrichment paradigm triggers release of serum-derived extracellular vesicles (EVs) that promote myelination/remyelination and reduce measures of oxidative stress [5, 6]. Analogous EVs can be produced *ex vivo* by stimulating bone marrow-derived dendritic cell cultures with interferon gamma (IFNγ), a pro-inflammatory cytokine that is phasically increased with environmental enrichment [7].

EVs secreted by IFNγ-stimulated dendritic cells (IFNγ-DC-EVs) are 30–300 nm in size (this represents a 30–150 nm population and a 150–300 nm population; see S1 Fig), show typical morphology by electron microscope imaging, and express surface markers ALG-2-interacting protein X (Alix) and cluster of differentiation 63 (CD63), as we have shown in a previous publication (see Pusic *et al*., 2014) [8]. Here, we characterize IFNγ-DC-EVs by immunoblotting for Alix and CD63, electron microscopy, and dynamic light scatter analysis (S1 Fig). These EVs promote myelination/remyelination, reduce oxidative stress, and preferentially enter oligodendrocytes in rat brain slice cultures. When compared to unstimulated dendritic cell EVs, IFNγ-DC-EVs contain higher levels of microRNAs involved in oligodendrocyte differentiation pathways, myelin production pathways and anti-inflammatory pathways (for all data related to the above, see Pusic *et al*., 2014) [8]. It is important to note that IFNγ-DC-EVs do not permanently elevate myelin levels in normal (uninjured) brain, as doing so could produce detrimental effects. In time-course studies, we have seen that increased myelin (as measured via myelin basic protein levels) is evident by three days post administration, peaks at five days, and returns to baseline levels by 12 days post administration [9]. However, it is likely that based on need (e.g., demyelinating disease) or increased neural activity (e.g., learning), increased myelin levels following IFNγ-DC-EVs treatment may be maintained. These results prompted us to suggest IFNγ-DC-EVs as a novel neurotherapeutic for neurodegenerative disorders involving demyelination and brain inflammation such as multiple sclerosis and migraine.

We went on to show that IFNγ-DC-EVs increase grey matter myelin [8], reduce markers of oxidative stress and inhibit spreading depression when nasally administered to rats [10]. Spreading depression is the likely cause of migraine aura and perhaps related pain. It is a well-established model of migraine that triggers increased oxidative stress and transient demyelination [11].

While we can infer that nasally administered IFNγ-DC-EVs enter the CNS through measurement of these functional effects, we have not yet directly tracked them through imaging studies. Determining the distribution of intranasally administered IFNγ-DC-EVs in the CNS is an important step toward therapeutic development.

Here, we aimed to demonstrate nasal delivery of functional IFNγ-DC-EVs. While the paths by which nasally administered EVs pass into the brain are not fully understood, if entry is rapid, as with nasally administered insulin-like growth factor-1, it is likely they travel through the nasal mucosal barrier with little impediment and are then distributed throughout the brain by bulk flow in the CSF [12]. Using IVIS imaging, we found that EVs could be detected in the rostral portion of the brain following intranasal administration. Explant imaging for biodistribution showed that they distributed throughout the CNS, but did not significantly accumulate in other organs. Consistent with prior evidence from *in vitr*o studies using slice cultures [8], IFNγ-DC-EVs were taken up by cells that were predominantly of oligodendrocyte lineage, and to a lesser extent microglia, after nasal administration to whole animals. Finally, a comparison of intranasal versus intravenous administration showed that, while EVs entered the CNS via both routes, intranasal administration allowed for more specific delivery.

Our results provide evidence that IFNγ-DC-EVs enter the brain, distribute throughout the parenchyma, and functionally deliver their contents. This data further supports use of IFNγ-DC-EVs as a novel therapeutic for neurodegenerative disorders that effect myelination.

## Materials and methods

### Extracellular vesicle preparation

EVs were prepared from rat bone marrow dendritic cells as previously described [6, 8, 13]. Briefly, bone marrow and stromal cells were isolated from femur and tibia of male Wistar rats and plated at a density of 10^6^ cells/mL in RPMI 1640 (#31800089, Invitrogen) containing 10% FBS (#16140071, Invitrogen) and 20 ng/mL of rat recombinant GM-CSF (#400–23, Peprotech) for differentiation into bone marrow derived dendritic cells. Medium was changed on day two and five, and cells in suspension harvested for replating on day seven. For generation of EVs, medium was prepared using exosome-depleted FBS (#EXO-FBSHI-50A-1, System Biosciences). Day seven cells were plated at 10^6^ cells/mL in medium alone (Unstimulated, or Unstim-DC-EVs) or with the addition of 500 U/mL IFNγ (#585-IF; R&D Systems) (IFNγ-DC-EVs). Three days later conditioned medium was harvested for Unstim-DC-EV and IFNγ-DC-EV isolation. EVs were isolated from conditioned culture medium using ExoQuick-TC (#EXOTC10A-1, System Biosciences). Quantification of EVs was performed by BCA assay (ThermoFisher). We recognize that a significant portion of the μg weight of our EV sample prepared using ExoQuick is likely to be non-active or excipient.

We believe excipient material in our samples is actually beneficial for two reasons. It may play a role as a carrier protein to reduce aggregation and aid in stability of samples, and it allows us to more accurately administer a systematic amount of EVs.

In practically all studies to date, EVs are isolated by one of a variety of methods that yield an ‘EV enriched’ preparation that likely represents a heterogenous mixture of exosomes (30–150 nm range), microvesicles (200–1000 nm range), apoptotic bodies and protein complexes [14, 15]. Analyzing our EVs using dynamic light scatter (*n* = 6) resulted in an average 1.51 ± 0.52% volume of particles in the 30–150 nm diameter “exosome” range. As our starting samples consisted of 100 μg protein (as determined by BCA) in 50μL sterile PBS, this corresponds to roughly 1.5 μg of EVs in the “exosome” size range. EVs (typically defined as 30-1000nm in diameter, though the largest vesicles observed in our samples were < 300 nm) make up an average 3.06 ± 1.13% volume, and would bring the μg estimate to roughly three μg of EVs in a 100 μg sample (S1 Fig).

Our work shows that this level of active EVs consistently produces a significant effect *in vitro* and *in vivo* after nasal delivery. IFNγ-DC-EV preparations are routinely screened for increased miR-219 using TaqMan MicroRNA Assays (rno-miR-219; #002077, Applied Biosystems), with U6 snRNA (#001973) and U87 snRNA (#001712) as endogenous controls. A significant (i.e., >2-fold) increase in miR-219 content in IFNγ-DC-EVs versus Unstim-DC-EVs is the criterion for use in subsequent experiments [8].

### Whole animal administration of EVs

All procedures involving animals were approved by the Institutional Animal Care and Use Committee at the University of Chicago and were conducted in accordance with the National Institutes of Health and Guide for the Care and Use of Laboratory Animals (2011) [16]. Rats were housed (two animals/cage) in static micro isolator cages with corn cob bedding and were provided with Enviro-Dri nesting material (Shepherd Specialty Papers) and nestlets (Anacore Corporation). Rats were maintained on a 12-hour light-dark cycle with controlled temperature and humidity in our Central Animal Facility. 2918 irradiated Teklad diet (Envigo) and acidified water were available *ad libitum*. All procedures were performed using aseptic techniques.

#### Nasal administration

Adult (262–475 g) male Wistar rats (Charles River Laboratories) were nasally administered EV preparations. Rats were placed in a fume hood with a heat lamp and thermo-regulator (TC-5000; Digi-Sense) to maintain temperatures at 37 ± 0.5°C. Isoflurane (Butler Schein Animal Health) anesthesia was delivered in oxygen via a nose cone (5% induction and 2–3% maintenance) with spontaneous respiration. Arterial oxygen tension was continuously monitored with a pulse oximeter (2500A VET; Nonin Medical, Plymouth, MN). Adequacy and consistency of anesthesia in spontaneously breathing animals was monitored by noting: 1) arterial oxygen tension that was kept between 95–100 mm Hg; 2) pink skin color in appendages; 3) regular respirations; and 4) absence of withdrawal to hind paw pinch [16, 17]. Animals were placed in a supine position, and 50 μL of EVs in phosphate buffered saline (PBS; 100 μg protein) were administered over a 20 minute period at a rate of five μL every two minutes to alternating nostrils [6, 13, 18, 19]. Sham animals were administered 50 μL of a vehicle (PBS, exosome-depleted culture medium/dye preparations, Unstim-DC-EVs or non-transfected IFNγ-DC-EVs as noted below in specific experiments). Animals were sacrificed and organs harvested as indicated below.

#### Intravenous administration

Lateral tail vein injections were utilized for intravenous administration of EVs. Briefly, rats were anesthetized with inhalational isoflurane and monitored as above for nasal administration. Animals were placed in a prone position and vein dilation was stimulated by briefly placing the tail in warm (30-35°C) water. A sterile 27 gauge needle was used to inject 200 μL of EVs in phosphate buffered saline (PBS; 100 μg protein) or equivalent sham control solution.

### EV staining and brain imaging strategies

#### mCherry EV transfection and imaging

Reporter gene mRNA for mCherry (CleanCap mCherry, #L-7203, TriLink BioTechnologies) was transfected into IFNγ-DC-EVs using Lipofectamine MessengerMax (#LMRNA003, Invitrogen) according to the manufacturer’s protocol. For tracking experiments, 200 μg of isolated IFNγ-DC-EVs were transfected with 10 μg of mCherry mRNA, re-isolated using ExoQuick-TC and resuspended in 55 μL of sterile PBS. Five μL of the EV solution was applied to hippocampal slice cultures to confirm transfection and validate anti-mCherry immunostaining (S2 Fig). Opti-MEM transfection supernatant was administered to slice cultures as an additional control to ensure that transfected EVs and not lipofectamine-encapsulated mCherry mRNA was responsible for mCherry mRNA delivery to cells. Rats were nasally administered 50 μL of transfected EVs once a day for two days. Sham controls consisted of rats nasally administered untransfected IFNγ-DC-EVs. Brains and spinal cord were harvested six hours after the final dose. Brains were sliced into 3–4 mm thick coronal slices using a Rat Brain Matrix (#RBMC-300C; Kent Scientific), then immersion fixed for two hours in cold 2% paraformaldehyde-PBS. Fixed brain slices were cryopreserved in 20% sucrose.

Sections (14 μm) were cut in a cryostat (#CM3050S, Leica) and directly mounted to slides before post-fixation for five minutes in ice cold methanol. Slides were incubated for one hour at room temperature in blocking buffer (1X PBS, 5% normal serum, 0.05% Tween 20), then incubated overnight at 4°C with primary antibodies. Primary antibodies used were anti-mCherry (#AB0040-200, Sicgen; 1: 250) and anti-CNPase (#AB9342, Millipore-Sigma; 1:500), followed by the appropriate Alexa Fluor secondary antibody (#A21467 Chicken anti-Goat IgG 488; #A28175 Goat anti-mouse IgG 488, ThermoFisher; 1: 1000) for 1 hour at room temperature. Slides were coverslipped with ProLong Gold Antifade Reagent (#9071S, Molecular Probes).

Images were acquired with an SP8 confocal microscope [using a 63x objective N.A. 1.40] (Fig 2A–2C and S2 Fig) at one μm steps and presented as z-stack projection images. Additional images (Fig 2D) were acquired using a sensitive CCD digital imaging system consisting of a QuantEM-512SC camera (Photometrics), electronic shutter (Lambda SC Smart Shutter; Sutter instruments), and a 100 watt Hg light on a DMIRE2 inverted microscope (Leica) at using a 20x objective N.A. 0.30. Images were subsequently processed using ImageJ.

#### IVIS imaging

EVs were labelled with Xenolight DiR (#125964; PerkinElmer), a lipophilic near infrared dye that stably stains cytoplasmic membranes with negligible dye transfer between cells. 10 mL of conditioned medium was incubated with two μM dye and EVs were isolated using ExoQuick-TC. Residual dye was removed by spin filtering (Exosome Spin Columns, MW 3000, #4484449; ThermoFisher). DiR-EVs were resuspended in 50 μL sterile PBS. The same procedure was done with 10 mL of Exo-Free (non-conditioned) medium for sham controls. Sham controls were prepared in this way to account for the possibility that A) spin filters do not efficiently remove free dye, or B) that other media components are labelled by Xenolight DiR. Any false positive fluorescent signal produced by either of these confounds is therefore accounted for during data analysis. Rats were anesthetized with isoflurane in oxygen (as described above) and their heads and backs shaved with electric clippers before chemical depilation (Nair hair removal lotion). Animals were then nasally administered DiR-EVs or sham media control solutions. Prior to imaging in an IVIS Spectrum 2000 imaging system (PerkinElmer), animals were anesthetized by injection with xylazine/ketamine. For *ex vivo* imaging, animals were nasally administered DiR-EVs or sham control solutions and sacrificed at specified times for collection of brain, liver, kidney, spleen and lung. Brains were subsequently dissected into three mm thick sections using Rat Brain Matrix (#RBMC-300C and RBMC-300S; Kent Scientific). A sagittal slice from left hemisphere and serial coronal sections of the right hemisphere were imaged. Analysis was performed using LivingImage software (PerkinElmer). To ensure uniformity of measurements, ROIs were automatically generated, and defined as a minimum 10% of peak pixel intensity. Where this was not possible (i.e., no fluorescence signal detected in controls), a stereotypical ROI of equivalent area to that automatically generated in corresponding experimental animals was used.

### Immunoblotting

Three days after nasal administration of IFNγ-DC-EVs or Unstim-DC-EVs (sham), animals were anesthetized with progressive exposure to 100% carbon dioxide and decapitated. Brains were rapidly removed and protein was extracted from samples of neocortex tissue using RIPA buffer with complete c (#4693132001; Roche). Protein expression was evaluated using standard SDS-PAGE and immunoblotting protocols. Equal amounts of protein (15 or 40 μg) were run on 4–15% TGX mini gels (#4561085; Bio-Rad) and transferred onto PVDF membranes (#1704156; Bio-Rad). Primary antibodies used were PDGFRα (#NBP2-67025; Novus Biologicals) at 1:5000, and ELOVL7 (#NBP1-93926; Novus Biologicals) at 1:750. Secondary antibody, horseradish peroxide conjugated anti-mouse or anti-rabbit (#A5278 and #A0545; Millipore-Sigma) was used at a concentration of 1:1000 and blots were then visualized via chemiluminescence on a Bio-Rad Chemidoc Imaging System. Analysis was performed using Image Lab software (Bio-Rad). All protein was normalized to vinculin (#ab129002, Abcam), with both primary and secondary antibody concentrations of 1:1000.

### Statistical procedures

Data were analyzed using SigmaPlot (v.12.5; Systat Software, Inc.). All data were subject to normality testing (*p*-value to reject: 0.05), equal variance testing (*p*-value to reject: 0.05), and power (1-β: > 0.8). Mean control data in each experiment were scaled to 1.00 with all subsequent parameters scaled proportionally to better allow inter-experiment comparisons. All experimental groups consisted of biological replicates of *n* ≥ 3. Pairwise comparisons were made with the Student’s *t*-test and multiple comparisons done via ANOVA plus *post hoc* Holm-Sidak testing.

CorelDRAW X7 (Corel Corporation) and then Adobe Photoshop CC 2017 (Adobe System, Inc.) were used to construct final figure images. All images were processed in pairs (i.e., experimental versus sham) and equivalently treated [20].

## Results

### IFNγ-DC-EVs distribute throughout the brain following nasal administration

#### IVIS imaging of nasally administered EVs

**Fig 1:**
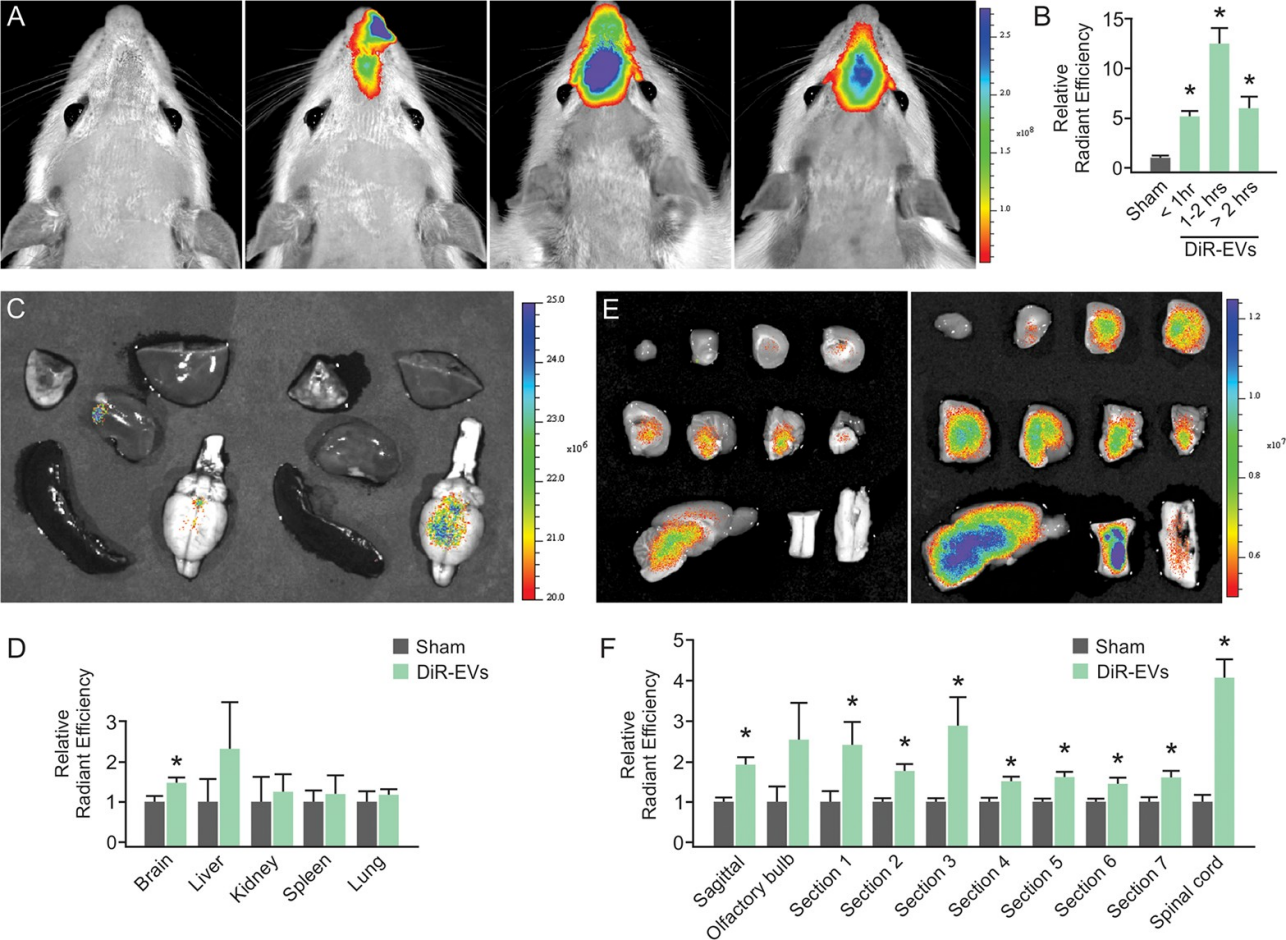
Biodistribution of IFNγ-DC-EVs after nasal delivery. (A) Representative low-power images of Xenolight DiR-labelled IFNγ-DC-EVs (DiR-EVs) fluorescence in the rostral portion of the nose/brain (Left-to-right: sham, < one hour post nasal administration of DiR-EVs, one-to-two hours post nasal administration, > two hours post nasal administration). Sham controls consisted of exosome-free (non-conditioned) media prepared in the same manner as conditioned media for labelling of EVs. (B) Quantification of radiant efficiency shows significantly (*p = 0.040, <0.001 and 0.025, respectively) higher fluorescence in the forebrain area of animals nasally administered DiR-EVs versus sham. (C) Imaging of organs (Top row: lung, kidney, liver; bottom row: spleen and brain) was performed for whole brain (therapeutic target) and liver, kidney, spleen (clearance/toxicity) and lung (for error in nasal delivery) at one hour post nasal administration. Representative images of Sham (Left) and DiR-EV (Right) organs are shown. (D) Quantification of radiant efficiency showed significantly (*p = 0.018) increased signal in nasal administration DiR-EV whole brain, but not in other organs. To better visualize DiR-EV uptake, explanted brains were serially dissected into three mm sections. (E) Representative images of Sham (Left) and DiR-EV (Right) after nasal administration are shown, with coronal brain sections (1–8), a sagittal section to the lower left and two regions of upper (left) and mid (right) cervical spinal cord to the lower right. (F) Quantification of radiant efficiency showed significantly (*p = 0.003–0.048) increased signal in all brain sections except olfactory bulb (*n* = 5-7/group; C-F).

IFNγ-DC-EVs were dyed with Xenolight DiR (DiR-EVs) for IVIS imaging. We detected significantly (*p* = <0.001–0.04) higher fluorescence in the rostral portion of the brain in animals nasally administered DiR-EVs compared to sham controls (Fig 1A). Specific values were (Fig 1B): [*p* = 0.04; sham (1.00 ± 0.24), <1 hour post nasal administration (5.15 ± 0.65), *p* < 0.001; 1–2 hours post nasal administration (12.40 ± 1.79), *p* = 0.025; >2 hours post nasal administration (5.84 ± 1.19)]. Imaging of explanted organs included whole brain (therapeutic target), liver, kidney and spleen (clearance) and lung (for error in nasal delivery) (Fig 1C). While fluorescence signal was significantly (*p* = 0.018) higher in the brains of animals nasally administered DiR-EVs (1.48 ± 0.11) versus sham controls (1.00 ± 0.14), fluorescence levels for all other organs were not significantly different (*n* = 6-8/group) (Fig 1D). To better visualize spatial distribution, brains were dissected into a single three mm thick sagittal section from the left hemisphere, and the right hemisphere was serially sectioned into three mm coronal slices (Fig 1E). Quantification of radiant efficiency showed significant (*p* = 0.002–0.027) increased fluorescence in all brain sections except for the olfactory bulb (Fig 1G). Specific values were: Sagittal section [*p* = 0.002; sham (1.00 ± 0.14), DiR-EV (1.92 ± 0.21)], Section 1 [*p* = 0.048; sham (1.00 ± 0.27), Dir-EV (2.42 ± 0.66)], Section 2 [*p* = 0.003; sham (1.00 ± 0.10), DiR-EV (1.76 ± 0.20)], Section 3 [*p* = 0.013; sham (1.00 ± 0.11), DiR-EV (1.5 ± 0.14)], Section 4 [*p* = 0.006; sham (1.00 ± 0.11), DiR-EV (1.62 ± 0.17)], Section 5 [*p* = 0.012; sham (1.00 ± 0.11), DiR-EV (1.52 ± 0.15)], Section 6 [*p* = 0.017; sham (1.00 ± 0.11), DiR-EV (1.47 ± 0.14)], Section 7 [*p =* 0.027; sham (1.00 ± 0.16), DiR-EV (1.61 ± 0.18)], Spinal cord [*p* = 0.014; sham (1.00 ± 0.21), DiR-EV (4.12 ± 1.42)].

### Nasally administered IFNγ-DC-EVs functionally delivery their contents to brain cells

#### IFNγ-DC-EVs can be transfected with/deliver mCherry mRNA

**Fig 2:**
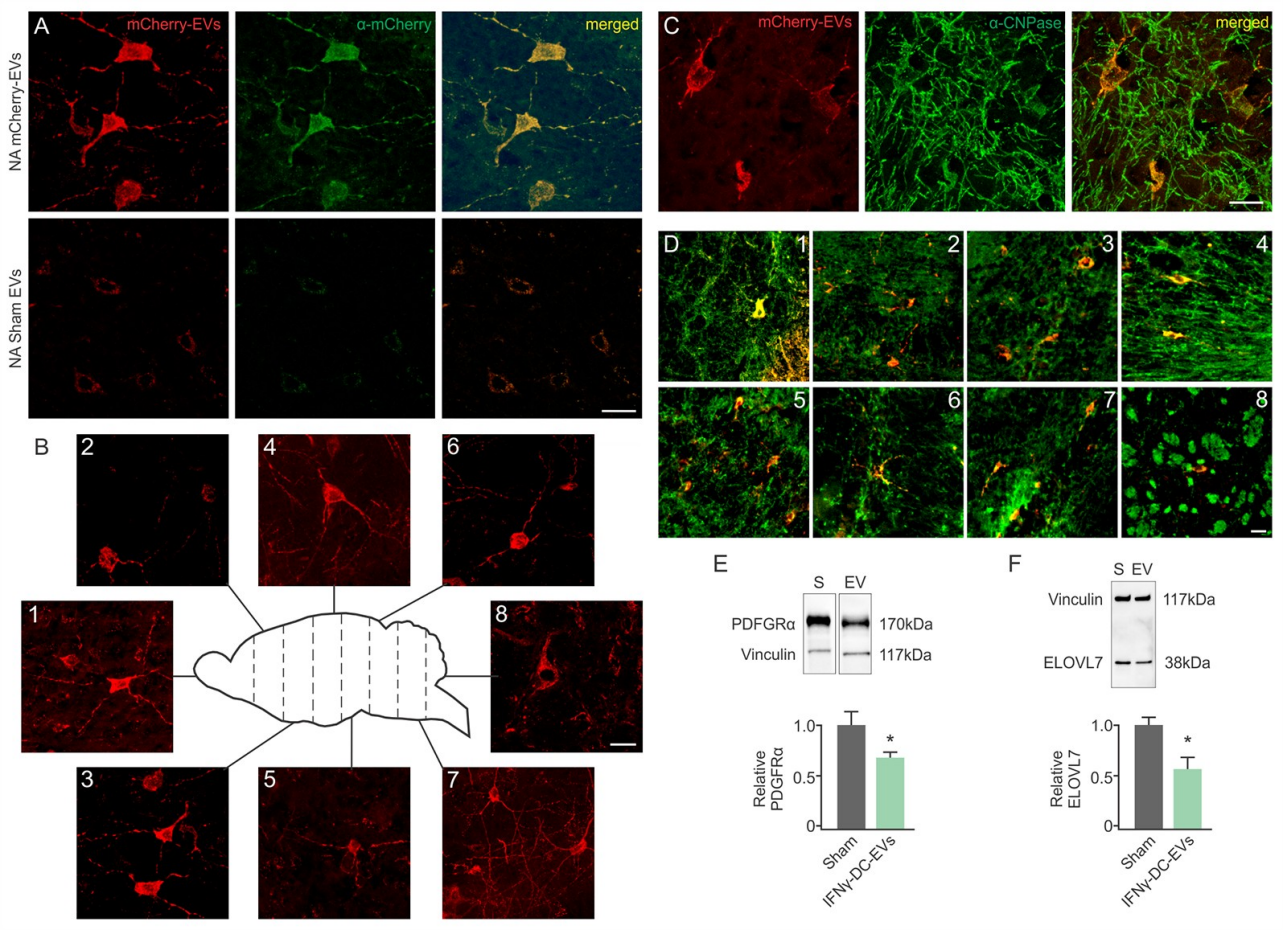
Nasally administered IFNγ-DC-EVs deliver their contents to brain cells. IFNγ-DC-EVs were transfected with reporter mCherry mRNA. (A) Nasal delivery of transfected EVs (NA mCherry-EV) confirmed EV delivery of functional mCherry mRNA to brain cells. Representative images show mCherry fluorescence (red), anti-mCherry immunostaining (green) and merged image (yellow) to confirm mCherry expression in cells. Animals that were nasally administered untransfected EVs (Sham) showed no mCherry expression/staining. Scale bar = 25 μm. (B) Representative merged images (B1-8) show widespread cellular distribution in the CNS two days after nasal administration of mCherry mRNA transfected IFNγ-DC-EVs. Images were taken from brain sections represented in the schematic. A subset of mCherry-positive cells colocalize with CNPase, indicating that they are oligodendrocytes. (C) Representative high power images of mCherry expression (red) in cells that co-stained (yellow) for CNPase (green). Scale bar = 25 μm. (D) Similarly, stained representative merged images (D1-8) show oligodendrocyte co-staining throughout the CNS, with numbers corresponding to brain areas in schematic (B). Scale bar = 20 μm. (*n* = 5-7/group; A-D). To confirm that IFNγ-DC-EVs delivered cargo to oligodendrocytes, protein expression levels of known miR-219 mRNA targets expressed in oligodendrocyte lineage cells were quantified via immunoblot. Three days after nasal delivery of IFNγ-DC-EVs or Unstim-DC-EVs [Sham(S)], levels of (E) PDGFRα, and (F) ELOVL7 were significantly (*p = 0.040 and 0.038 respectively) reduced in the brains of animals that received IFNγ-DC-EVs. (*n* = 3-6/group).

IFNγ-DC-EVs were transfected with mCherry mRNA prior to nasal administration (mCherry-EVs). Transfection was confirmed *in vitro* using hippocampal slice cultures, and expression of mCherry protein verified by immunohistological staining with an anti-mCherry antibody (S2 Fig). Nasal administration of mCherry-EVs induced expression of mCherry protein in CNS cells, as verified by staining with anti-mCherry (Fig 2A, top row). This staining was not evident in the brains of rats nasally administered untransfected IFNγ-DC-EVs (Fig 2A, bottom row) (*n* = 5-7/group).

#### mCherry-transfected IFNγ-DC-EVs distribute throughout the CNS following nasal administration

After nasal administration of mCherry-EVs, mCherry-positive cells were found in coronal sections taken along the rostrocaudal axis as far caudal as the brainstem and cervical spinal cord (*n* = 5–7 from above group). Images are shown for all sections (Fig 2B; 1–8).

#### mCherry expressing cells co-localize with CNPase

Co-staining for oligodendrocytes [2’,3’-Cyclic-nucleotide 3’-phosphodiesterase (CNPase)], neurons [microtubule-associated protein (MAP2)], astrocytes [glial fibrillary acidic protein (GFAP)], and microglia [ionized calcium binding adaptor molecule 1 (IBA1)] showed qualitatively that mCherry expressing cells largely co-localized with CNPase (S3 Fig). Punctate co-localization within IBA1 positive cells was also observed. Double positive cells were found in coronal sections taken along the rostrocaudal axis (Fig 2C and 2D) (*n* = 5–7 from above group).

#### IFNγ-DC-EVs deliver their contents to oligodendrocyte lineage cells

In a previous study, we reported that IFNγ-DC-EVs contain high levels of miR-219, which is required for production of mature oligodendrocytes via regulation of multiple mRNA targets involved in the differentiation process [8]. As an additional confirmation that nasally administered IFNγ-DC-EVs are taken up by cells of the oligodendrocyte lineage, we performed immunoblots for known targets of miR-219. We selected a target involved in oligodendrocyte maturation, as well as one involved in maintenance of myelin by mature cells. Specific targets were: PDGFRα (Fig 2E), the receptor for a mitogen that keeps oligodendrocyte precursor cells in a non-differentiated proliferative state; and ELOVL7 (Fig 2F), which regulates lipid metabolism and redox homeostasis [21, 22]. Three days after nasal administration, protein levels of both targets were significantly (*p* = 0.038 and 0.040) lower in IFNγ-DC-EV-treated compared to sham (Unstim-DC-EV) treated animals (*n* = 3-6/group). Specific values were: PDGFRα [*p* = 0.040; sham (1.00 ± 0.14), EV (0.68 ± 0.06)], ELOVL7 [*p* = 0.038; sham (1.00 ± 0.15), EV (0.57 ± 0.11)]. This further supports our hypothesis that nasally administered IFNγ-DC-EVs target oligodendrocytes.

### IVIS imaging shows differences in distribution of IFNγ-DC-EVs following nasal versus intravenous administration

**Fig 3:**
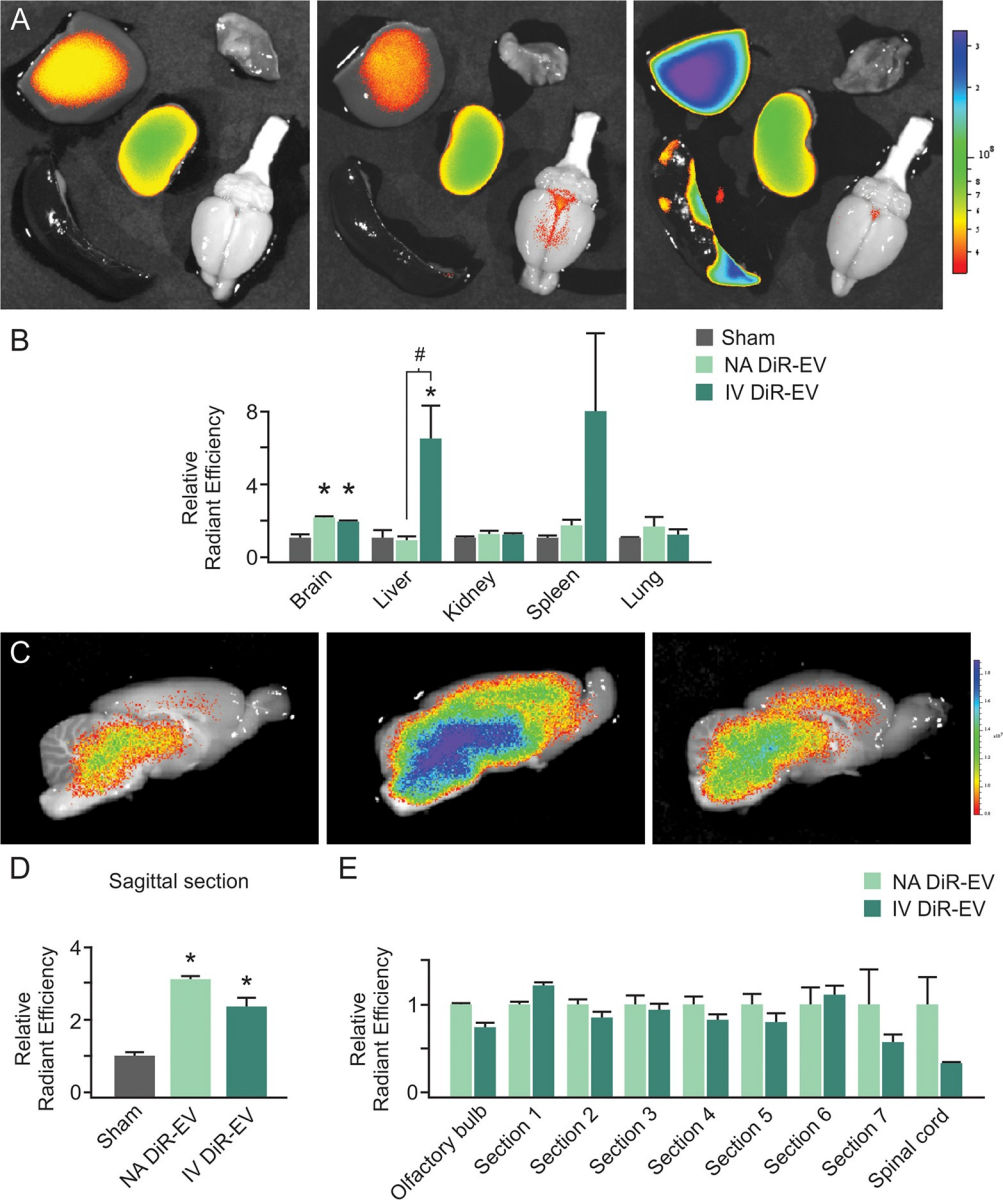
Comparison of intranasal versus intravenous delivery of IFNγ-DC-EVs. (A) Representative images of explanted organs at one hour post-administration of Xenolight DiR-labelled EVs. (Top row: liver and lung; middle: kidney; bottom row: spleen and brain) [Left to right: Sham, nasally administered (NA) DiR-EVs, intravenously delivered (IV) DiR-EVs]. Sham controls consisted of intravenously administered Xenolight DiR-labelled exosome-free (non-conditioned) medium prepared in the same manner as conditioned media for labelling of EVs. (B) Quantification shows significantly higher (*p <0.001) signal in nasally administered and intravenously delivered DiR-EV brains, and significantly higher (*p = 0.027) signal in intravenously delivered DiR-EV liver. There was also a significant difference (#*p* = 0.050) between liver DiR-EV fluorescence in intravenously versus nasally treated animals. No other organ measured showed significant differences. (C) Representative images of 3 mm thick sagittal sections of brain [Left to right: sham, nasally administered DiR-EVs, intravenously delivered DiR-EVs]. (D) Quantification of sagittal sections showed significantly (*p < 0.001) increased signal in both nasally administered and intravenously delivered DiR-EV administered animals versus sham. (E) Quantification of additional brain areas showed no significant difference in CNS distribution between delivery routes (Sections 1–7 defined as in Fig 1). (*n* = 3-4/group; A-E).

Finally, we compared the efficacy of intravenous versus intranasal delivery. IFNγ-DC-EVs were stained with Xenolight DiR dye as before (DiR-EVs) and administered either nasally or intravenously (via tail vein injection). Animals were sacrificed and IVIS imaging was performed at one hour post administration, consistent with our previously performed *ex vivo* experiments. Whole organ imaging showed differences in delivery to the brains and livers of animals that received DiR-EVs via nasal administration or intravenous delivery compared to sham animals that received intravenous Xenolight DiR-treated unconditioned medium (Fig 3A). Radiant efficiency was significantly higher in DiR-EV treated animals compared to sham (*p* = 0.005 for nasal administration, *p* = 0.007 for intravenous delivery). Specific values for whole brains were: sham (1.00 ± 0.20), nasal administration (2.18 ± 0.18) and intravenous delivery (1.96 ± 0.12). Radiant efficiency was significantly higher in the liver of animals intravenously administered DiR-EVs versus that of both sham animals (*p* = 0.035) and animals nasally administered DiR-EVs (*p* = 0.048) animals. There was no significant increase in liver radiant efficiency above sham levels in animals nasally administered DiR-EVs. Specific values were: sham (1.00 ± 0.40), nasal administration (0.88 ± 0.29) and intravenous delivery (6.40 ± 2.00). No significant differences were observed in any other organs (Fig 3B). Next, we sectioned brains as before to examine the spatial distribution of DiR-EVs in the brains of nasal administration versus intravenous delivery animals (Fig 3C). In sagittal sections, both nasal administration and intravenous delivery of DiR-EVs resulted in significantly (*p* < 0.001 in both instances) higher radiant efficiency versus sham. Specific values were: sham (1.00 ± 0.13), nasal administration (3.14 ± 0.11) and intravenous delivery (2.34 ± 0.23) (Fig 3D). When normalized to shams, there were no significant differences in radiant efficiency in serially sectioned brains following nasal versus intravenous treatment (*n* = 3-4/group) (Fig 3E).

## Discussion

The work presented here expands on previous studies from our lab demonstrating functional changes in the CNS following nasal administration of IFNγ-DC-EVs. Three days after nasal administration to naïve rats, IFNγ-DC-EVs stimulate an increase in myelin basic protein levels [8]. Nasal administration of IFNγ-DC-EVs also inhibits spreading depression, the likely cause of migraine aura and a well-established experimental model of migraine. This latter effect is likely due to their impact on oxidative status. Following nasal administration of IFNγ-DC-EVs, brain expression of iNOS (a microglial M1 polarization product) and protein carbonyl levels (a biomarker of oxidative damage) are significantly reduced, suggesting a reduction in oxidative stress [10]. Despite the evidence of effective nasal delivery afforded by these functional measurements, we felt it was important to directly demonstrate CNS entry.

Nasal administration is a non-invasive technique for delivering treatments to the brain that allows rapid absorption and quick onset of action. Use of the nasal approach may reduce systemic side effects, and even allow for self-administration of therapeutics. Although the actual mechanism by which this occurs is still unclear, there is evidence that both small and large molecules can cross the nasal mucosa through a paracellular or transcellular route, then be transported into the brain via olfactory and trigeminal nerve pathways [23, 24]. These pathways bypass the blood brain barrier and provide a direct connection from the olfactory neuro-epithelium in the nasal cavity to the brain [25]. The dominant mechanism by which a substance enters the brain is determined by its physicochemical properties, formulation, and delivery device [26].

There are numerous examples in the literature of EV delivery to the CNS via nasal administration. These studies, like our previous work, show evidence of successful nasal delivery by measuring functional outcomes. Others additionally showed imaging evidence of CNS uptake. A variety of EV types and imaging strategies have been used. For example, Zhuang et al., (2011) encapsulated drugs in EVs derived from a murine lymphoma cell line, and labelled them with DiR for imaging [27]. They found rapid (within one hour) delivery to the brain following nasal administration, and report selective uptake by microglial cells. Haney *et al*., (2015) loaded DiL-stained macrophage EVs with catalase for nasal administration to a mouse model of Parkinson’s disease, and saw uptake by neuronal cells four hours later [28]. Betzer *et al*., (2017) labelled mesenchymal stem cell EVs with glucose-coated gold nanoparticles and used *in vivo* neuroimaging to track their entry following nasal administration to a mouse stroke model [29]. Long *et al*., (2017) showed that PKH26-stained mesenchymal stem cell EVs are taken up by neurons and microglia within six hours of nasal administration to a mouse model of epilepsy [30]. Perets *et al*., (2018) used MAESTRO whole brain imaging to visualize PKH26-stained mesenchymal stem cell EVs 24 hours after nasal administration to a mouse model of neuroinflammation, and report uptake by neurons [31]. In short, the utility of intranasal administration as an EV delivery route has been well documented. However, these studies also illustrate that the targeting ability of EVs depends on their source. EV surface composition varies based on their derivation, and their uptake may be additionally impacted by the physiologic state of the recipient cell. Stimulation state can also impact the properties of EVs derived from the same cell type, as we have previously reported that small microvesicles (presumed to be exosomes) from IFNγ-stimulated or unstimulated dendritic cells are taken up by CNS cells at different rates. When applied to hippocampal slice cultures, IFNγ-DC-EVs preferentially entered oligodendrocytes (72%), whereas EVs from unstimulated dendritic cells showed no oligodendrocyte specificity (7%) and instead preferentially entered astrocytes (63%) [8]. Both types of EVs were taken up by microglia at a similar rate. Thus, confirming the ability of our specific EVs to enter the CNS via the nasal delivery route and target oligodendrocytes is a significant step towards determining the utility of IFNγ-DC-EVs as a therapeutic for demyelinating disorders.

IFNγ-DC-EVs were labelled with Xenolight DiR and IVIS imaging was used to track nasally administered EVs. DiR-EVs were detected in the rostral portion of the brain starting at 30 minutes post nasal administration. This rapid entry is consistent with literature reporting significant levels of administered molecules [32–34] and EVs [27, 29] in the parenchyma within an hour. While we observed fluorescent signal in the brains of sham animals, this was likely because sham treatments consisted of Xenolight DiR-treated unconditioned medium. Unconditioned medium contains serum proteins and possibly other nanoparticles (despite our use of exosome-depleted serum) that could be stained with Xenolight DiR. Use of this method to prepare sham treatments allows us to account for any false positive fluorescence resulting from nasal delivery of Xenolight DiR-labelled extraneous material or remaining free dye. The foci of highest radiant efficiency was seen further caudally over time. By over two hours post nasal administration (2.5 to 5+ hours) radiant efficiency began to diminish, suggesting uptake of EVs and/or dispersion of fluorescently labelled vesicle membranes as they integrate with cellular membranes. This observation is inconsistent with data from others showing sustained presence of EV fluorescence for upwards of 24 hours post nasal administration. It is possible that this discrepancy is due to our use of wildtype adult rats in this study, whereas most other reports in the literature utilize mouse models of neurological diseases in their imaging studies. An adult Wistar rat skull ranges from 0.5–1 mm thick, whereas an adult mouse skull is approximately 0.2 mm thick [35], and inflammation is known to impact EV uptake [36, 37]. In fact, Betzer *et al*., (2017) reported that EVs were retained near ischemic lesion sites in injured brains, but cleared from uninjured brains by 24 hours after nasal administration [29]. Imaging methodology and the properties of the dye or particle used to label EVs may also contribute to discrepancies.

Due to concerns about whether fluorescent signal from DiR-EVs could penetrate through the brain and skull of an adult rat, we performed *ex vivo* imaging of explanted organs including whole brain (therapeutic target), liver, kidney and spleen (clearance) and lung (for error in nasal delivery). Results showed significantly increased radiant efficiency in whole brains of DiR-EV treated animals, but no significant differences in any other organ, suggesting that nasal delivery reduces systemic spread. To better visualize spatial distribution, brains were dissected into a single three mm thick sagittal section from the left hemisphere, and the right hemisphere was serially sectioned into three mm slices. Quantification of radiant efficiency showed significantly increased fluorescence in all brain sections except for the olfactory bulb.

To determine whether IFNγ-DC-EVs are taken up by CNS cells and deliver functional contents, EVs were transfected with mCherry mRNA prior to nasal delivery. mCherry expression, confirmed via immunostaining with an anti-mCherry antibody, was found in cells throughout the brain as far back as the cervical spinal cord. Co-localization with CNPase showed that mCherry expressing cells were predominantly of the oligodendrocyte lineage. CNPase expression is highest during differentiation of immature pre-oligodendrocytes, and is also expressed by mature oligodendrocytes [38]. Punctate mCherry fluorescence was also occasionally seen in microglial cells, but we did not observe co-localization in neurons or astrocytes. This is consistent with our previously reported *in vitro* data showing that quantum dot-conjugated IFNγ-DC-EVs applied to hippocampal slice cultures preferentially enter oligodendrocytes, and to a lesser extent microglia, as opposed to neurons and astrocytes [8].

We have previously reported that IFNγ-DC-EVs contain higher levels of miRNA species involved in oligodendrocyte differentiation and anti-inflammatory pathways when compared to EVs from unstimulated dendritic cells [8, 39]. Notably, they contain high levels of miR-219, which plays a central role in formation/maintenance of myelin and is necessary for oligodendrocyte precursor cell maturation through inhibition of negative regulators of differentiation [21, 40]. miR-219 is highly conserved, and homologues are found in a wide variety of vertebrate species [41]. Overexpression of miR-219 is sufficient to induce differentiation *in vitro* [42], and *in vivo* [22], and many studies have explored its role in promoting myelin repair and functional recovery in animal models of multiple sclerosis [43, 44] and other demyelinating injuries [45]. Interestingly, Osorio-Querejta *et al*., (2020) have shown that nasal administration of EVs enriched with miR-219a-5p more efficiently induced oligodendrocyte precursor cell differentiation and improve clinical scores in an EAE model than miRNA-loaded liposomes and polymeric nanoparticles, despite the latter containing higher levels of miR-219-5p [46].

We have shown that treating primary oligodendrocyte precursor cells with IFNγ-DC-EVs promotes their differentiation to the same extent as treatment with triiodothyronine (T3) or transfection with a miR-219 mimic [8]. To produce this result, it would be necessary for IFNγ-DC-EVs to be taken up by oligodendrocyte precursor cells and transfer biologically active miR-219. Thus, as an additional means to determine IFNγ-DC-EV entry into oligodendrocyte lineage cells, we looked for evidence of translational repression and/or mRNA degradation. We measured protein levels of several miR-219 targets involved in oligodendrocyte differentiation/myelination in the brains of animals that received nasally administered IFNγ-DC-EVs versus animals that received nasally administered Unstim-DC-EVs.

PDGFRα is expressed on oligodendrocyte precursor cells, and is the receptor for PDGF, a mitogen that promotes proliferation and inhibits differentiation [47]. A decrease in PDGFRα would encourage oligodendrocyte maturation. ELOVL7 is a fatty acid elongation factor that is necessary for myelin maintenance, but can lead to lipid accumulation and demyelination if overexpressed in oligodendrocytes [40]. Reduced expression of the protein products of these mRNA targets confirms that nasally administered IFNγ-DC-EVs deliver their cargo (including miR-219) to cells of the oligodendrocyte lineage, and may modulate their maturation by targeting multiple steps in the differentiation pathway. This is of particular interest, as oligodendrocyte precursor cells are present but unable to mature into myelinating cells in multiple sclerosis, perhaps in part due to deficient miR-219 in lesions [48].

Finally, we compared the efficacy of intravenous versus intranasal delivery, as administration route is known to be a factor in determining the outcomes of immune and therapeutic responses [49, 50]. Explant imaging of organs revealed increased fluorescence intensity in the livers of rats intravenously administered DiR-EVs versus those nasally administered DiR-EVs. Quantification of other organs did not rise to significance. These data suggest that while both intravenous and nasal routes of administration deliver IFNγ-DC-EVs to the brain, the nasal administration route provided enhanced CNS specificity. This is consistent with data from other groups showing that certain types of naturally produced EVs can be intravenously administered to cross the intact blood brain barrier [51–53]. Macrophage-derived EVs express lymphocyte function-associated antigen 1 (LFA-1), which interacts with intercellular adhesion molecule 1 (ICAM-1) on the blood brain barrier to facilitate uptake [36]. Interestingly, Yuan and colleagues found that ICAM-1 is upregulated with neuroinflammation, resulting in enhanced EV uptake during pathological conditions, suggesting that some EVs may not cross an intact blood brain barrier. In future work, it will be valuable to characterize IFNγ-DC-EV membrane proteins, to determine which surface receptors may be involved in their ability to cross the nasal mucosa, the blood brain barrier and enter oligodendrocytes.

Of relevance to our work, is a study by Qu *et al*., (2018) demonstrating that blood EVs naturally target the brain through transferrin-transferrin receptor interaction between blood-borne EVs and blood brain barrier cells [54]. Murine bone marrow-derived dendritic cell lines contain high levels of transferrin receptor, supporting the theory that dendritic cell-derived EVs can enter the brain via intravenous delivery. However, a direct comparison of nasal delivery versus intravenous administration of DiR-labelled IFNγ-DC-EVs revealed a key difference. While we observed fluorescence in the CNS one hour after administration by both routes, intravenous administration resulted in higher levels of accumulation in off-target organs, notably in the liver. This implies that nasal administration is an appropriate delivery route for more precisely targeting substances to the CNS while minimizing the potential for off-target effects. These data are supported by prior studies showing that nasal administration of drug-loaded EVs caused minimal delivery to the liver, and may account for the favorable toxicity profile of nasally delivered EVs [27, 55], and are consistent with studies showing that intranasal administration led to more specific CNS delivery than intravenous administration [28, 29, 31].

In summary, the work outlined above shows that IFNγ-DC-EVs can be rapidly delivered to CNS cells via nasal administration, without significant accumulation in off-target organs. Importantly, this is the first demonstration that nasally administered IFNγ-DC-EVs intrinsically target oligodendrocytes without being modified to express oligodendrocyte-specific binding proteins. We also provide evidence that IFNγ-DC-EVs may modulate oligodendrocyte maturation by regulating multiple steps in the differentiation pathway. Taken together, data presented here provide further support for the administration of IFNγ-DC-EVs via the nasal delivery route as a potential therapeutic to improve remyelination.

## Supporting information

S1 Fig IFNγ-DC-EV characterization. EV isolation was confirmed by (A) Immunoblot for surface markers CD63 and Alix, by (B) electron microscopy (scale bar, 50 nm), and (C) via dynamic light scatter analysis. The mean value and standard error for dynamic light scatter data is shown for (Left) particles in the 30–150 nm “exosome” range, and (Right) all EVs.
(TIF)

S2 Fig *In vitro* confirmation of IFNγ-DC-Exo transfection with mCherry mRNA. Representative images of hippocampal slice cultures treated with (A) transfected IFNγ-DC-EVs (mCherry-EVs) or (B) Opti-MEM transfection supernatant (transfection control). Images show successful EV transfection and subsequent expression of mCherry (red) in recipient cells. The specificity of mCherry expression was confirmed using an anti-mCherry antibody (green). Merged image (yellow) shows high degree of co-localization. Scale bar = 200 μm.
(TIF)

S3 Fig Cell specific staining shows that mCherry positive cells are predominantly oligodendrocytes. (A) Representative images show cell-specific immunofluorescence (top row, green), mCherry fluorescence (middle, red) and merged images (bottom). Left to right: Neurons (anti-MAP2), astrocytes (anti-GFAP), oligodendrocytes (anti-CNPase) and microglia (anti-IBA1). Scale bar = 25 μm. (B) Magnified merged image of IBA1 staining. Arrows indicate punctate co-localization with mCherry. Scale bar = 15 μm.
(TIF)

S1 File (PDF)

S2 File (DOCX)
