# Supplementary figures and images for: IFNγ-stimulated dendritic cell extracellular vesicles can be nasally administered to the brain and enter oligodendrocytes
Source: PLoS One. 2021 Aug 13;16(8):e0255778. doi: 10.1371/journal.pone.0255778 (PMC8363003; doi:10.1371/journal.pone.0255778)

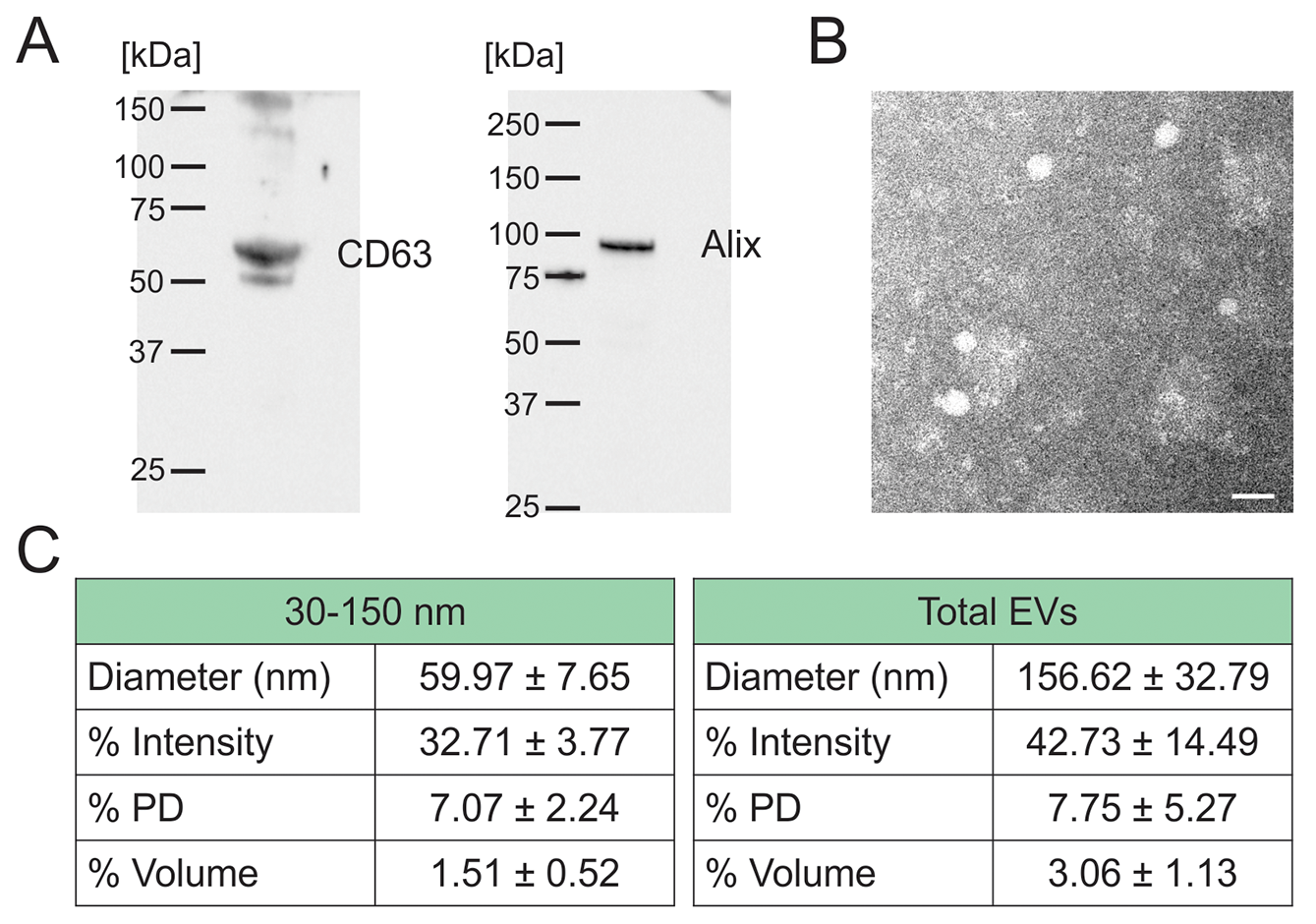

Supplement: S1 Fig — EV isolation was confirmed by (A) Immunoblot for surface markers CD63 and Alix, by (B) electron microscopy (scale bar, 50 nm), and (C) via dynamic light scatter analysis. The mean value and standard error for dynamic light scatter data is shown for (Left) particles in the 30–150 nm “exosome” range, and (Right) all EVs. (TIF) [file pone.0255778.s001.tif]

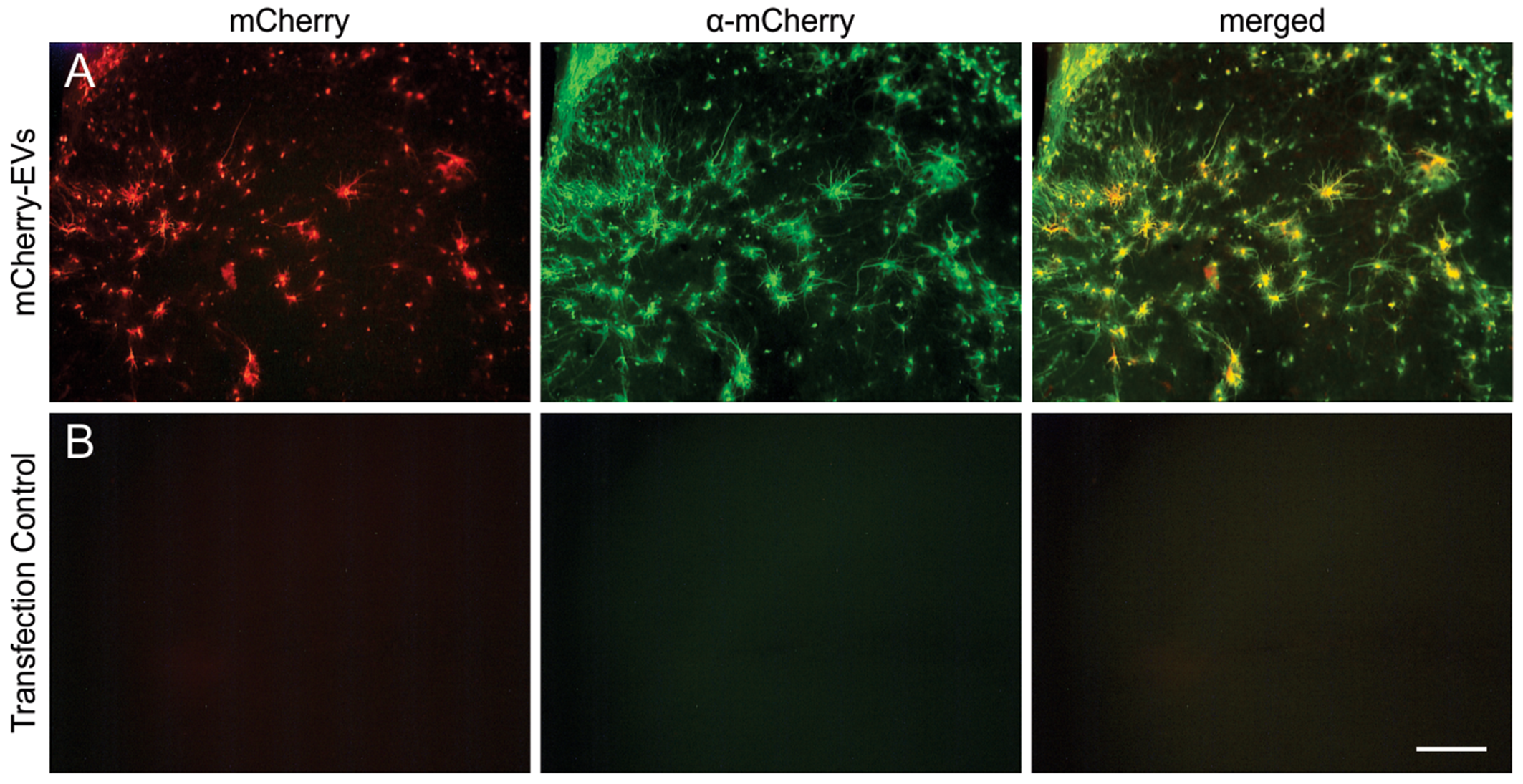

Supplement: S2 Fig — Representative images of hippocampal slice cultures treated with (A) transfected IFNγ-DC-EVs (mCherry-EVs) or (B) Opti-MEM transfection supernatant (transfection control). Images show successful EV transfection and subsequent expression of mCherry (red) in recipient cells. The specificity of mCherry expression was confirmed using an anti-mCherry antibody (green). Merged image (yellow) shows high degree of co-localization. Scale bar = 200 μm. (TIF) [file pone.0255778.s002.tif]

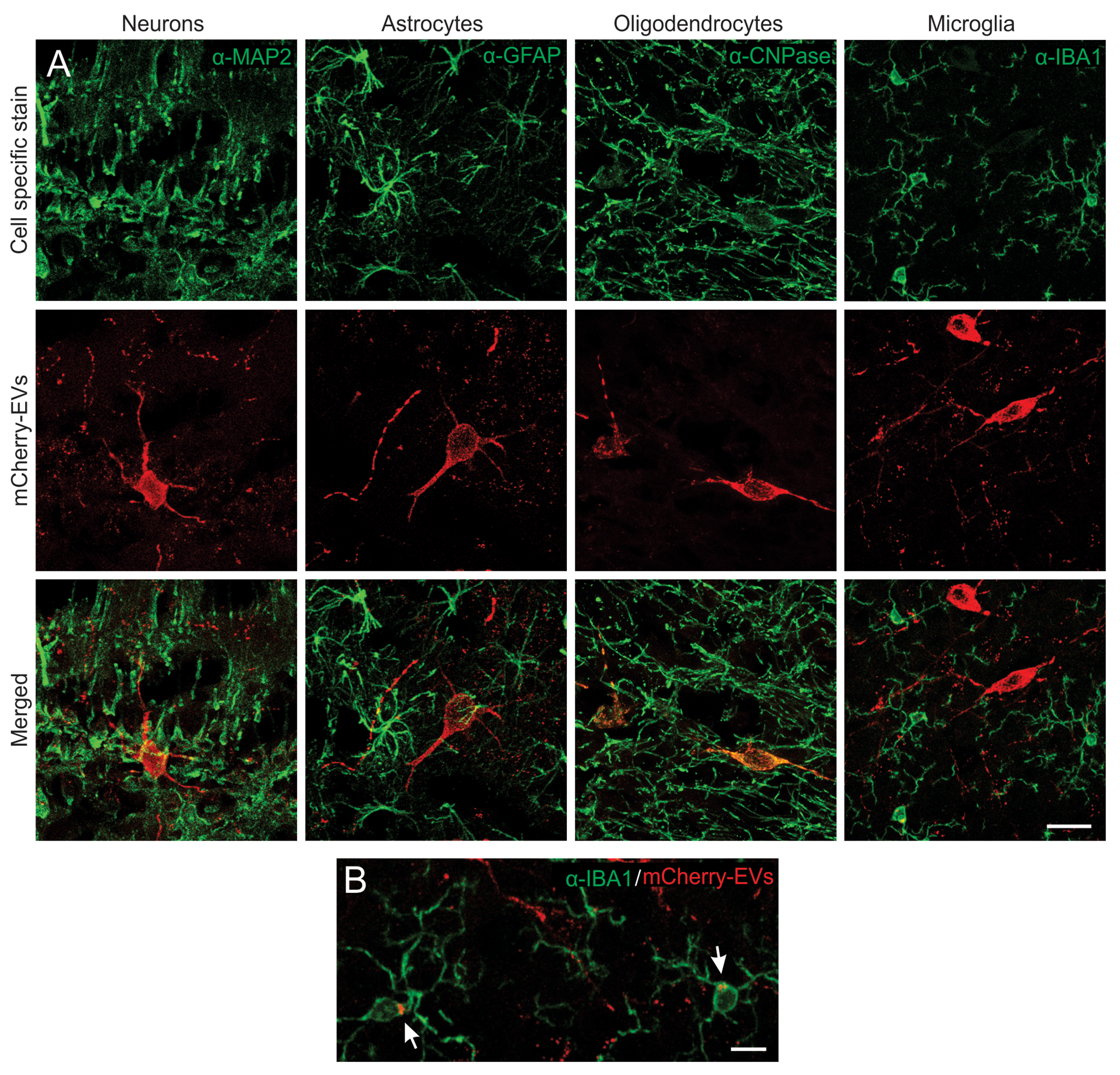

Supplement: S3 Fig — (A) Representative images show cell-specific immunofluorescence (top row, green), mCherry fluorescence (middle, red) and merged images (bottom). Left to right: Neurons (anti-MAP2), astrocytes (anti-GFAP), oligodendrocytes (anti-CNPase) and microglia (anti-IBA1). Scale bar = 25 μm. (B) Magnified merged image of IBA1 staining. Arrows indicate punctate co-localization with mCherry. Scale bar = 15 μm. (TIF) [file pone.0255778.s003.tif]
