# Supplementary material for: IFNγ-stimulated dendritic cell extracellular vesicles can be nasally administered to the brain and enter oligodendrocytes
Source: PLoS One. 2021 Aug 13;16(8):e0255778. doi: 10.1371/journal.pone.0255778 (PMC8363003; doi:10.1371/journal.pone.0255778)
Supplement: S1 File — (PDF) [file pone.0255778.s004.pdf]

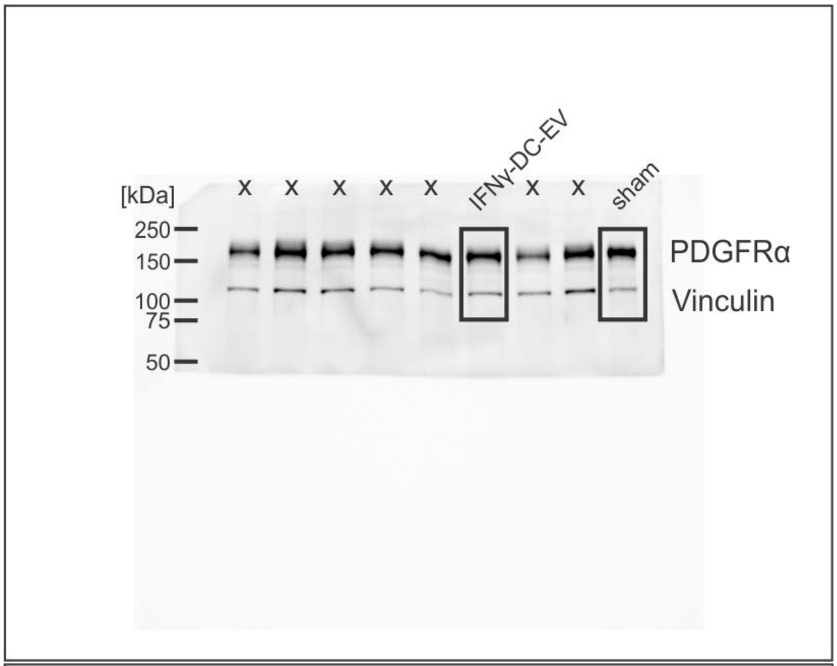

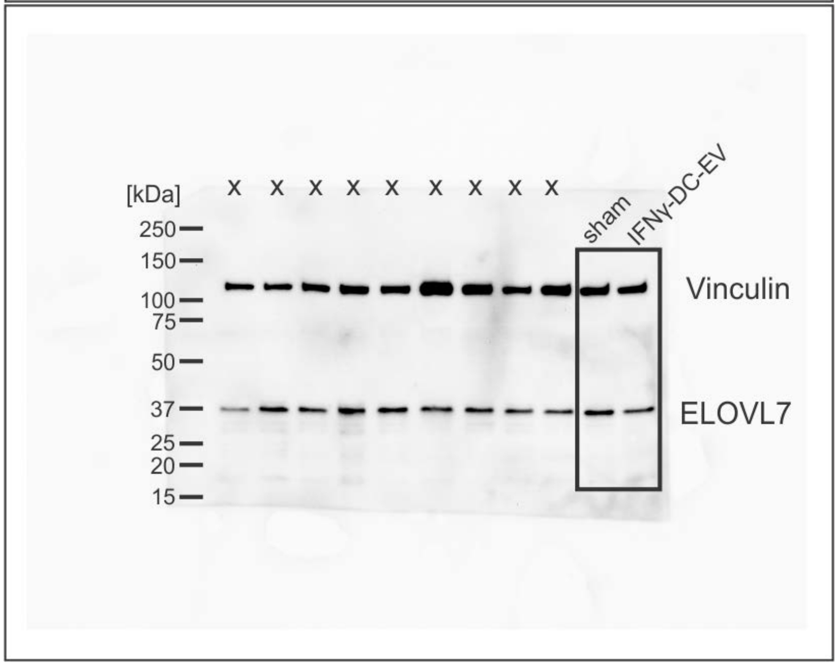

Raw images of immunoblots for PDGFRα and ELOVL7 (Fig 2E and 2F respectively). Equal amounts of protein (15 µg for PDGFRα or 40 µg for ELOVL7) were run on 4-15% TGX mini gels (#4561085; Bio-Rad) and transferred onto PVDF membranes (#1704156; Bio-Rad). Primary antibodies used were PDGFRα (#NBP2-67025; Novus Biologicals) at 1:5000, and ELOVL7 (#NBP1-93926; Novus Biologicals) at 1:750. Secondary antibody, horseradish peroxide conjugated anti-mouse or anti-rabbit (#A5278 and #A0545; Millipore-Sigma) was used at a concentration of 1:1000. Blots were developed using Pierce ECL Western Blotting Substrate (#32106; Thermo Scientific) and imaged on a Bio-Rad Chemidoc Imaging System. Analysis was performed using Image Lab software (Bio-Rad). All protein was normalized to vinculin (#ab129002, Abcam),
