# Supplementary material for: IFNγ-stimulated dendritic cell extracellular vesicles can be nasally administered to the brain and enter oligodendrocytes
Source: PLoS One. 2021 Aug 13;16(8):e0255778. doi: 10.1371/journal.pone.0255778 (PMC8363003; doi:10.1371/journal.pone.0255778)
Supplement: S2 File — (DOCX) [file pone.0255778.s005.docx]

**S2 File**

**EV characterization**

Recovery of EVs (including exosomes) was confirmed via immunoblotting for two well-characterized protein markers, CD63 (#MCA4754GA; BioRad) and Alix (#MCA2493; BioRad) following standard procedures. For electron microscopy, EVs were diluted 1:50 in 1% phosphotungstic acid and visualized under 300 kV using an FEI Tencai F30 transmission electron microscope with a Gatan CCD camera and Digital Micrograph software (University of Chicago Electron Microscopy Facility). Dynamic light scatter data was aquired on a DynaPro NanoStar instrument (Wyatt Technologies, University of Chicago Biophysics Core Facility). Three acquisitions of five s at 25ºC were collected for each sample (*n* = 6) and analyzed using Dynamics V7 software.

**Slice culture preparation**

Hippocampal slice cultures (350 μm) were prepared from P9-P10 rat pups as previously described. After 18 days *in vitro* (DIV) cultures were transferred to a serum-free medium, which does not activate microglia and does not contain horse serum-derived EVs, thus allowing for accurate assessment of the impact of EV treatments. Cultures were screened for viability by staining with Sytox (Invitrogen), a fluorescent cell death marker, and used when mature, at 21-35 DIV. Following IFNγ-DC-EV transfection with mCherry mRNA (see methods in main manuscript), five µL of the EV solution was applied to hippocampal slice cultures. Cultures were monitored every few hours to visualize development of mCherry expression, and fixed at 24 hours in cold 2% paraformaldehyde-PBS. mCherry expression was confirmed via immunostaining with an anti-mCherry antibody as described in the main manuscript. Images were acquired using a sensitive CCD digital imaging system consisting of a QuantEM-512SC camera (Photometrics), electronic shutter (Lambda SC Smart Shutter; Sutter instruments), and a Lumen 200 watt Xeon lamp (Prior) on a DMIRE2 inverted microscope (Leica) at using a 20x objective N.A. 0.30. Images were subsequently processed using ImageJ.

**mCherry imaging**

As per the main manuscript methods, rats were nasally administered 50 µL of transfected EVs once a day for two days. Brains and spinal cord were harvested six hours after the final dose. Brains were sliced into three-four mm thick coronal slices using a Rat Brain Matrix (#RBMC-300C; Kent Scientific), then immersion fixed for two hours in cold 2% paraformaldehyde-PBS. Fixed brain slices were cryopreserved in 20% sucrose.

Sections (14 µm) were cut in a cryostat (#CM3050S, Leica) and directly mounted to slides before post-fixation for five minutes in ice cold methanol. Slides were incubated for one hour at room temperature in blocking buffer (1X PBS, 5% normal serum, 0.05% Tween 20), then incubated overnight at 4ºC with primary antibodies. Primary antibodies used were anti-CNPase (#AB9342, Millipore-Sigma; 1:500), anti-MAP2 (#AB5622, Millipore-Sigma; 1:500), anti-IBA1 (#019-19741, Wako; 1:1000), and anti-GFAP (#sc-51908, Santa Cruz Biotechnologies; 1:1000) followed by the appropriate Alexa Fluor secondary antibody (#A28175 Goat anti-mouse IgG 488; #A11008 Chicken anti-rabbit IgG 488, ThermoFisher; 1: 1000) for one hour at room temperature. Images were acquired with an SP8 confocal microscope [using a 63x objective N.A. 1.40 (Fig. 2A-C)] at one μm steps and presented as z-stack projection images.
